# Novel potato plants with enhanced cadmium resistance and antioxidative defence generated after *in vitro* cell line selection

**DOI:** 10.1371/journal.pone.0185621

**Published:** 2017-10-02

**Authors:** Seyedardalan Ashrafzadeh, David W. M. Leung

**Affiliations:** School of Biological Sciences, University of Canterbury, Private Bag 4800, Christchurch, New Zealand; United States Department of Agriculture, UNITED STATES

## Abstract

It is of interest to apply plant tissue culture to generate plants resistant to toxic effects of cadmium (Cd) on plant growth. Callus cultures were initiated from leaf explants of micropropagated potato plantlets (*Solanum tuberosum* L., cv. Iwa) for *in vitro* selection comprising 18 different Cd treatments varying in Cd exposure timing and duration. Plantlets regenerated from two different lines of Cd-selected calli, L9 and L11, were found to exhibit enhanced resistance to 218 μM Cd compared to control (source plantlets for leaf explants used to initiate callus cultures for Cd resistance). In response to 218 μM Cd, L11 plantlets had lower levels of lipid peroxidation and hydrogen peroxide than control and L9 plantlets. In addition, antioxidative enzyme activities in L11 were generally higher than control. L11 also had a higher level of proline than control.

## Introduction

Cadmium (Cd) is a highly toxic trace element to plant growth [1, 2] and a potential human carcinogen [3]. Conventional plant breeding methods may be used to exploit natural variation in Cd resistance among different crop cultivars [4]. This involves costly labour-intensive field trials which can be problematic due to spatial heterogeneity of soil chemical and physical properties, and seasonal fluctuations [5,6].

*In vitro* plant breeding is another genetic improvement approach using somaclonal variants that might exhibit improved resistance to Cd found in plant cell and tissue cultures [7,8]. For *in vitro* selection, calli are often exposed to Cd as the stress factor or selecting agent during proliferation (subculture) or even from earlier (the callus induction stage) to select for Cd resistance [9]. Plants regenerated from the selected Cd-resistant calli have to be evaluated under *in vitro* conditions to validate whether the Cd-resistance was expressed at the whole plant level [7]. As *in vitro* screening is conducted under aseptic conditions, the results obtained would not be complicated by potential interferences/interactions with microorganisms in comparison with field trials. The Cd-resistant somaclonal variant plants are a novel genetic resource to aid the efforts to mitigate / manage Cd hazards to crops and environment [4, 10]. They can also be studied in comparison with the parent plants to uncover the underlying Cd resistance mechanisms or desirable attributes that might include enhanced tolerance, minimized uptake or translocation of the Cd [11,12].

Potato (*Solanum tuberosum* L.) is a staple crop species of the Solanaceae (nightshades) family. Worldwide, it is the fourth most cultivated crop after wheat, rice and maize with the production of around 323 million tonnes [13]. To the best of our knowledge, the present study was the first report of generating cadmium-resistant somaclonal variants of potato. The novel Cd-resistant somaclonal potato lines were selected from callus culture of the parent Iwa variety that is highly amenable to tissue culture manipulations [14]. Since Cd-induced oxidative stress is known to be associated with Cd-induced injuries to plants [1, 10], it was hypothesized that the Cd-resistant plants obtained from *in vitro* selection would be better protected against oxidative stress following Cd exposure than the parent plants (control). This study is also an important prerequisite before any somaclonal variants may be advanced for use in development of new Cd-resistant potato cultivars [15, 16].

## Materials and methods

### Basal culture medium and culture conditions

All the media were half-strength basal Murashige and Skoog (MS) medium [17] supplemented with 3% (w/v) sucrose, adjusted to pH 5.8, and solidified with 0.8% (w/v) agar. Afterwards, they were autoclaved at 121°C under 103 kPa for 20 minutes. All the calli and plantlets were grown inside 250-ml clear polycarbonate tissue culture jars (8.5 cm height and 6.5 cm diameter) in a growth room at 21 ±1°C under continuous lighting (Sylvania Gro-Lux lamps 36W).

### *In vitro* cell line selection

Micropropagated potato plantlets (*Solanum tuberosum* L. cv. ‘Iwa’) were obtained from the stock cultures at the University of Canterbury and propagated on medium A (Table 1) as described previously [18]. Callus cultures were initiated using leaf explants on medium B (Table 1: half-strength MS medium supplemented with 4.43 μM BA and 5.37 μM NAA) [19]. For *in vitro* selection at the callus induction stage, Cd was not added to the medium (medium B as described in Table 1) in the even numbered treatments, while the callus induction medium (medium B as described in Table 1) in the odd numbered treatments was supplemented with 27 μM Cd (Table 1). After three weeks of culture, the calli formed were isolated from explants and then grown on the callus proliferation medium (medium B as described in Table 1) supplemented with 109 μM Cd that was previously found to be sub-lethal to potato callus growth [19] in the different rounds of subculture. Depending on a particular treatment, the number of rounds of callus sub-culture ranged from two to ten but the duration of each round was the same (two weeks) (Table 2). After each round of subculture, non-necrotic (green) patches of calli were excised from the necrotic sections and then placed on fresh medium of the same composition for the next round of subculture. Each treatment was repeated four times, and there were 50 explants or pieces of calli in each treatment.

**Table 1:** Different plant tissue culture media used in this study.

Medium code	Composition	Purpose/Use
A	half-strength basal Murashige and Skoog (MS) medium [17] supplemented with 3% (w/v) sucrose, but no plant growth regulators added	• Micropropagation of potato stock plants and plantlets obtained from *in vitro* cell line selection • Rooting of shoots after plant regeneration experiments
B	Half-strength basal MS medium supplemented with 4.43 μM BA and 5.37 μM NAA [19]	• Callus initiation from leaf explants of micropropagated plantlets • Callus proliferation or subculture of callus isolated from the leaf explants
C	half-strength basal MS medium supplemented with 2.88 to 14.43 μM GA_3_ alone or in combination with different concentrations of BA (2.21 to 8.87 μM) or zeatin (2.28 to 9.12 μM (see also Table 3)	Experiments for plant regeneration from potato callus

**Table 2:** Summary of eighteen treatments for selection of Cd-resistant potato calli.

Treatment number	27 μM Cd in C.I.^1^ medium		Sub-culture rounds^2^ on C.P.^3^ medium containing sub-lethal Cd level (109 μM)
1	Sub-inhibitory		2
2	0		2
3	Sub- inhibitory		3
4	0		3
5	Sub- inhibitory		4
6	0		4
7	Sub- inhibitory		5
8	0		5
9	Sub- inhibitory		6
10	0		6
11	Sub- inhibitory		7
12	0		7
13	Sub- inhibitory		8
14	0		8
15	Sub- inhibitory		9
16	0		9
17	Sub- inhibitory		10
18	0		10

^1^: Callus induction (C.I.) medium supplemented with 27 μM Cd (see Medium B in Table 1)

^2^: Each round of subculture was two weeks.

^3^: Callus proliferation (C.P.) medium supplemented with109 μM Cd (see Medium B in Table 1)

### Plant regeneration

**Fig 1:**
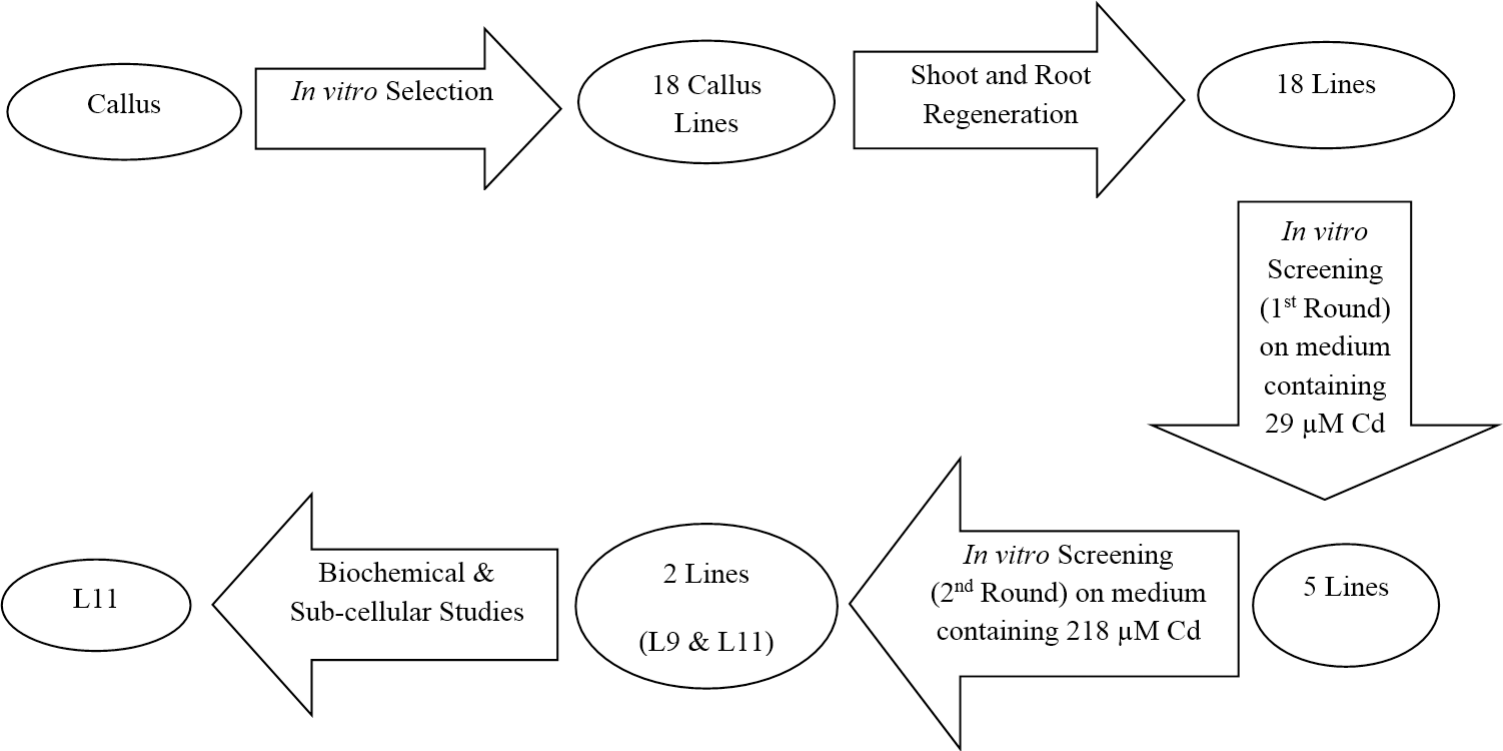
A schematic summary of the sequence of major steps towards generation and characterization of Cd-resistant potato lines.

Control calli (which were induced and proliferated on Cd-free Medium B, Table 1) were cultured on half-strength basal MS medium supplemented with 2.88 to 14.43 μM GA_3_ alone or in combination with different concentrations of BA (2.21 to 8.87 μM) or zeatin (2.28 to 9.12 μM) (Table 1; also see Table 3). There were 20 pieces of calli in each of the three replicates of a treatment. The optimized medium was used for shoot regeneration from calli obtained from the 18 different *in vitro* selection treatments (see Table 2 and Fig 1). Shoot regeneration frequency was evaluated for each treatment and all the regenerated shoots were kept on the optimized medium for 4 weeks. To initiate root formation, the shoots (at least 5 cm long with four developed leaves) were firstly separated from the surrounding calli and were then cultured on half-strength basal MS medium without any plant growth regulator (Medium A as described in Table 1). Root formation and development were assessed after 2 weeks of culture. There were three replicates each comprising 20 shoot cuttings.

**Table 3:** Shoot regeneration responses of potato calli to different combinations and concentrations of GA_3_, BA and zeatin.

Growth regulators (μM)				
BA	Zeatin	GA_3_	Days to shoot initiation	Shoot regeneration frequency %
0	0	11.54	-	0^f^
	0	14.43	-	0^f^
4.43	0	11.54	26–30	23.3±1.1^c^
6.65	0	11.54	26–30	26.6±0.7^c^
8.87	0	11.54	26–30	83.1±1.2^a^
0	4.56	11.54	28–33	17.3±1.8^d^
0	6.84	11.54	27–33	16.3±1.9^d^
0	9.12	11.54	27–33	77.4±1.1^ab^
4.43	0	14.43	26–30	56.2±1.4^b^
6.65	0	14.43	26–30	21.0±2.2^cd^
8.87	0	14.43	26–30	11.6±2.0^de^
0	4.56	14.43	29–32	9.6±1.5^e^
0	6.84	14.43	29–32	9.0±1.1^e^
0	9.12	14.43	27–31	9.7±0.4^e^

Data collected after 5 weeks of culture. Means within a column having the same letter are not significantly different by Duncan’s multiple range test (p<0.05).

The plantlets regenerated from each treatment were maintained as an individual line and was given the number of the treatment. The plantlets were propagated every 4 weeks by culturing nodal explants in fresh Cd-free half-strength basal MS medium (Medium A as described in Table 1). Depending on the length of Cd-resistant callus selection procedure in each treatment, the regenerated plantlets were subjected to at least three subculture rounds (~3 months in total), before they were harvested to undergo further *in vitro* screening for Cd resistance.

### *In vitro* screening for Cd resistance after plant regeneration

To identify plant lines with enhanced Cd resistance, their growth was first compared (1^st^ round of screening, Fig 1) to that of control plants in the presence of a relatively low, non-lethal Cd concentration (29 μM). For each plant line, shoot segments (3 cm each) with three leaves were excised from the middle portion of plantlets. Afterwards, the 18 excised segments (one from each of the putative somaclonal variant plant lines) were randomly cultured in six jars (three segments in each jar) containing 50 mL of basal half-strength MS medium (Medium A as described in Table 1) supplemented with 29 μM CdCl_2_ for 10 days. One control segment prepared in the same way as the variant lines was also co-cultured with the other segments from the plant lines in each jar.

The 2^nd^ round of screening was carried out with five lines selected based on the findings in the 1^st^ screening round (Fig 1). Like the 1^st^ round, shoot segments (3 cm each) with three leaves were excised from the middle portion of the plantlets of each line. The five excised segments as well as one control segment prepared in the same way as the variant lines were co-cultured in a jar containing 50 mL of half-strength basal MS medium (Medium A as described in Table 1) supplemented with 218 μM CdCl_2_ (the Cd concentration that was previously found to be lethal to potato plantlets, unpublished results) for 20 days. This was carried out with six replicates.

At the end of both screening rounds, the plantlets were handled and harvested very carefully to minimize root breakages during plantlet removal from the agar-gelled medium. The residual agar adhered to the roots was washed off using Milli-Q ultrapure water (Merck Millipore, Massachusetts, USA) before measuring the shoot and root lengths. Also, the number of newly developed leaves in each plantlet was counted. In the second screening round, visible injury symptoms including necrosis or chlorosis of each plantlet were also recorded. Afterwards, the fresh weights of the shoots and roots were determined separately. The shoot and root dry weights were also determined following drying in an oven (CONTHERM–CON8050) at 80°C for 48 hours.

### Biochemical characterization

Plantlets from each of the lines 9 and 11 as well as control stock plants that were cultured in the same jar with them were harvested after 7 days of culture on 50 mL half-strength basal MS medium supplemented with 0, 29 and 218 μM CdCl_2_. This was carried out with four replicates. The roots and leaves were separated for the biochemical analyses (see below).

#### Lipid peroxidation and hydrogen peroxide level

Lipid peroxidation was determined by measuring malondialdehyde (MDA) production in potato tissues and analyzed using the thiobarbituric acid (TBA) method [20]. Briefly, 0.5 g (by fresh weight) of roots or leaves was ground in liquid nitrogen using a mini pestle and mortar and then homogenized in 2.5 mL of ice-cold 0.1% (w/v) trichloroacetic acid (TCA). The homogenate was centrifuged at 12,000 g for 15 min at 4°C. Subsequently, 4 mL of 20% (w/v) TCA supplemented with 0.5% TBA was added to each 1 mL aliquot of the supernatant. Afterwards, the mixture was heated at 95°C for 30 min, cooled quickly on an ice-bath, and centrifuged for 15 min at 12,000 g. Finally, the absorbance was measured at 532 nm and then it was corrected by subtracting the absorbance at 600 nm (molar absorptivity of 155 mM^-1^ cm^-1^ was used in determination of MDA concentration).

After extracting 0.5 g (fresh weight, of roots or shoots), hydrogen peroxide (H_2_O_2_) level was determined according to Singh et al. [21]. Briefly, 0.5 g fresh weight of roots or leaves was ground in liquid nitrogen using a mini pestle and mortar and then homogenized in 5 ml of ice-cold 0.1% (w/v) trichloroacetic acid (TCA). The homogenate was centrifuged at 12,000 g for 15 min at 4°C. Afterwards, 0.5 ml of the supernatant was added to 0.5 ml of 10 mM potassium phosphate buffer (pH 7), and 1 mL of 1 mM potassium iodide (KI). Hydrogen peroxide content was measured by reading the supernatant absorbance at 390 nm (BIO RAD-Smartspec^TM^ Plus spectrophotometer).

#### Antioxidative enzymes

Enzyme extraction was carried out according to a modified method of Martins et al. [22]. One gram (fresh weight) roots or leaves were ground in liquid nitrogen using a mini pestle and mortar, and homogenized in 1 mL of 50 mM potassium phosphate buffer (pH 7.4) containing 0.1 mM Na_2_EDTA, and 2% (w/w) insoluble polyvinyl polypyrrolidone (PVP). The homogenate was then centrifuged at 12,000 g for 15 min at 4°C. Afterwards, the supernatant was filtered using VIVASPIN 500 (5000 MWCO) ultrafiltration units. Extraction of ascorbate peroxidase (APX) was carried out separately using the same method, except addition of 5 mM ascorbate to the potassium phosphate buffer. The activities of the antioxidative enzymes were determined according to the following procedures.

Superoxide dismutase (SOD; EC 1.15.1.1) activity was assayed according to the method of Martins et al. [22]. The enzyme activity was defined as the enzyme quantity required to inhibit the reduction of ferricytochrome-C by 50 percent, per min and mL. Catalase (CAT; EC 1.11.1.6) activity was determined according to the method of Martins et al. [22], by measuring a decrease in absorbance at 240 nm for 2 min in 50 mM phosphate buffer (pH 7.0) containing 10 mM of hydrogen peroxide. The enzyme activity was defined as the consumption of l μmol H_2_O_2_ per min. The effect of cadmium on guaiacol peroxidase (GPX; EC 1.11.1.7) activity was quantified as described by Ranieri et al. [23]. One unit of enzyme activity was defined as that which brought about a change of 0.01 absorbance unit per minute [23]. Ascorbate peroxidase (APX; EC 1.11.1.11) was determined according to the method of Sharma and Dubey [24]. One unit activity of APX is defined as the amount of enzyme which can oxidize 1 μM ascorbic acid per minute. Glutathione reductase (GR; EC 1.6.4.2.) activity was determined according to Phang et al. [25].

#### Proline content

Free proline content was determined according to Bates et al. [26]. Proline concentration was determined using a calibration curve and expressed as μMol proline mg^-1^ FW.

### Statistical analysis

All the experiments were carried out at least twice and every independent treatment had a minimum of three replicates. Data were subjected to one-way analysis of variance (ANOVA, p≤0.05) followed by comparison of the means using Duncan's multiple range test at 5% level of significance. The statistical program used was SPSS, version 19.0. The statistical analyses were applied to the data obtained from the following experiments: (i) plant regeneration trials; (ii) differences in growth parameters of the initial 18 plant lines regenerated from Cd-resistant calli to 29 μM Cd; (iii) differential growth responses of five selected potato lines to 218 μM Cd; and (iv) levels of reactive oxygen species, antioxidative enzyme activities and proline in the leaves and roots of control, and two selected plant lines exposed to 0, 29 and 218 μM Cd for 7 days.

## Results

### *In vitro* cell line selection and plant regeneration

The highest shoot initiation frequency (more than 75%) was found when ‘Iwa’ potato calli (control calli that had not been exposed to Cd-containing medium) were cultured on half-strength basal MS medium containing 11.54 μM GA_3_ in combination with 8.87 μM BA or 9.12 μM zeatin (Table 3). The first sign of shoot induction was observed slightly earlier (1 to 3 days) in the calli cultured on the medium supplemented with BA than zeatin. Therefore, half-strength basal MS medium supplemented with 11.54 μM GA_3_ + 8.87 μM BA was chosen for plantlet regeneration from Cd-selected calli (Table 3). Calli that survived from the final rounds in each of the 18 different *in vitro* cell line selection treatments were transferred to the optimized shoot regeneration medium at different times due to the different durations of treatments (ranging from one to five months). Root formation from control shoots (regenerated from calli that were not exposed to Cd) and those regenerated from Cd-selected calli occurred equally well on half-strength MS medium without addition of any plant growth regulator (PGR).

The frequency of shoot regeneration from Cd-selected calli was inversely correlated with the duration of Cd exposure (during callus induction and/or proliferation stages) in which they were selected (Table 1). The shoot induction frequency (83%) from calli selected after short-term Cd exposure including treatments 1–6 was not statistically different from that in control calli (not exposed to Cd) (Table 4). Prolonged Cd exposure of calli (for more than 5 months, treatment 18, Table 4) curtailed shoot regeneration frequency by about 30% compared to shoot regeneration frequency in calli selected on Cd-containing medium for a shorter duration (for example, treatment 1, Table 4).

**Table 4:** Shoot regeneration in eighteen treatments for selection of Cd-resistant potato calli.

Treatment number	27 μM Cd in C.I.^1^ medium	Sub-culture rounds^2^ on C.P.^3^ medium containing sub-lethal Cd level (109 μM)		Shoot regeneration frequency^4^
1	Sub-inhibitory		2	84.6±1.4^a^
2	0		2	82.1±1.0^a^
3	Sub- inhibitory		3	81.4±1.2^a^
4	0		3	82.2±0.9^a^
5	Sub- inhibitory		4	80.13±0.9^a^
6	0		4	78.8±1.3^ab^
7	Sub- inhibitory		5	75.7±2.1^b^
8	0		5	78.3±1.9^ab^
9	Sub- inhibitory		6	73.0±1.2^b^
10	0		6	70.4±1.7^bc^
11	Sub- inhibitory		7	70.3±1.7^bc^
12	0		7	68.2±2.2^c^
13	Sub- inhibitory		8	74.6±1.3^b^
14	0		8	65.7±1.5^c^
15	Sub- inhibitory		9	62.9±2.0^cd^
16	0		9	60.4±2.1^d^
17	Sub- inhibitory		10	60.5±1.1^d^
18	0		10	53.1±1.0^e^

^1^: Callus induction (C.I.) medium supplemented with 27 μM Cd (see Medium B in Table 1)

^2^: Each round of sub-culture was two weeks.

^3^: Callus proliferation (C.P.) medium supplemented with109 μM Cd (see Medium B in Table 1)

^4^. Shoot regeneration frequency with different letters was significantly different (p<0.05).

The plantlets obtained in each of the 18 treatments (Table 4) were regarded herewith as an individual variant line of the ‘Iwa’ cultivar (18 plant lines in total). They were named as L1 to L18 corresponding to the *in vitro* Cd selection treatments of the calli (1 to 18). Micropropagation in half-strength basal MS medium (Medium A as described in Table 1) was found to be useful for maintenance of the plantlets of all the variant lines.

### *In vitro* growth evaluation of plantlets regenerated from Cd-selected calli

In the first screening round, five plant lines namely L3, L6, L7, L9 and L11 exhibited significantly stronger growth abilities than the control in the presence of 29 μM (a low, non-lethal Cd concentration, Table 5). However, among them, L11 showed the best performance with respect to the chosen parameters, followed by L9 and L7 which both had almost the same growth abilities. With respect to almost all the parameters scored, the other 13 lines were similar to those of control plants (Table 4). Furthermore, the plantlets of the screened lines and the control did not show any visible injury symptoms and almost all of them seemed healthy after ten days of culture.

**Table 5:** Differential responses of 18 Cd-selected potato lines after 10 days of culture in the presence of 29 μM Cd in relation to vegetative growth (root length, shoot length and number of new leaves formed per plantlet).

Line	Root length(cm)	Shoot length(cm)	Number of new leaves formed	
Control	3.4±0.65^cd^	4.0±0.21^c^	1.3±0.12^e^	
1	3.4±0.43^cd^	4.1±0.34^c^	1.2±0.21^e^	
2	3.2±0.73^d^	4.2±0.11^c^	1.4±0.02^e^	
3	9.9±0.10^b^	6.4±0.56^b^	6.9±0.04^b^	
4	3.9±0.31^c^	4.4±0.07^c^	1.7±0.08^de^	
5	3.3±0.37^cd^	4.3±0.24^c^	1.1±0.10^e^	
6	10.2±0.22^b^	6.0±0.43^b^	6.0±0.14^c^	
7	11.1±0.17^a^	7.9±0.66^ab^	7.3±0.09^ab^	
8	3.0±0.48^d^	4.6±0.28^bc^	2.2±0.20^d^	
9	11.0±0.93^a^	8.0±0.33^ab^	7.4±0.12^ab^	
10	3.1±0.12^cd^	4.2±0.44^c^	1.9±0.18^d^	
11	11.3±0.14^a^	8.5±0.18^a^	7.9±0.04^a^	
12	3.1±0.19^cd^	4.2±0.22^c^	1.5±0.11^de^	
13	3.2±0.91^cd^	4.1±0.67^c^	1.5±0.32^de^	
14	3.4±0.80^cd^	4.4±0.90^c^	1.2±0.41^e^	
15	3.5±0.13^cd^	4.2±0.66^c^	1.4±0.07^e^	
16	3.1±0.59^cd^	4.1±0.10^c^	1.6±0.60^de^	
17	3.2±0.12^cd^	4.1±0.12^c^	1.4±0.44^e^	
18	3.0±0.09^d^	4.2±0.21^c^	1.2±0.56^e^	

Means within a column having the same letter are not significantly different by Duncan’s multiple range test (p<0.05).

**Fig 2:**
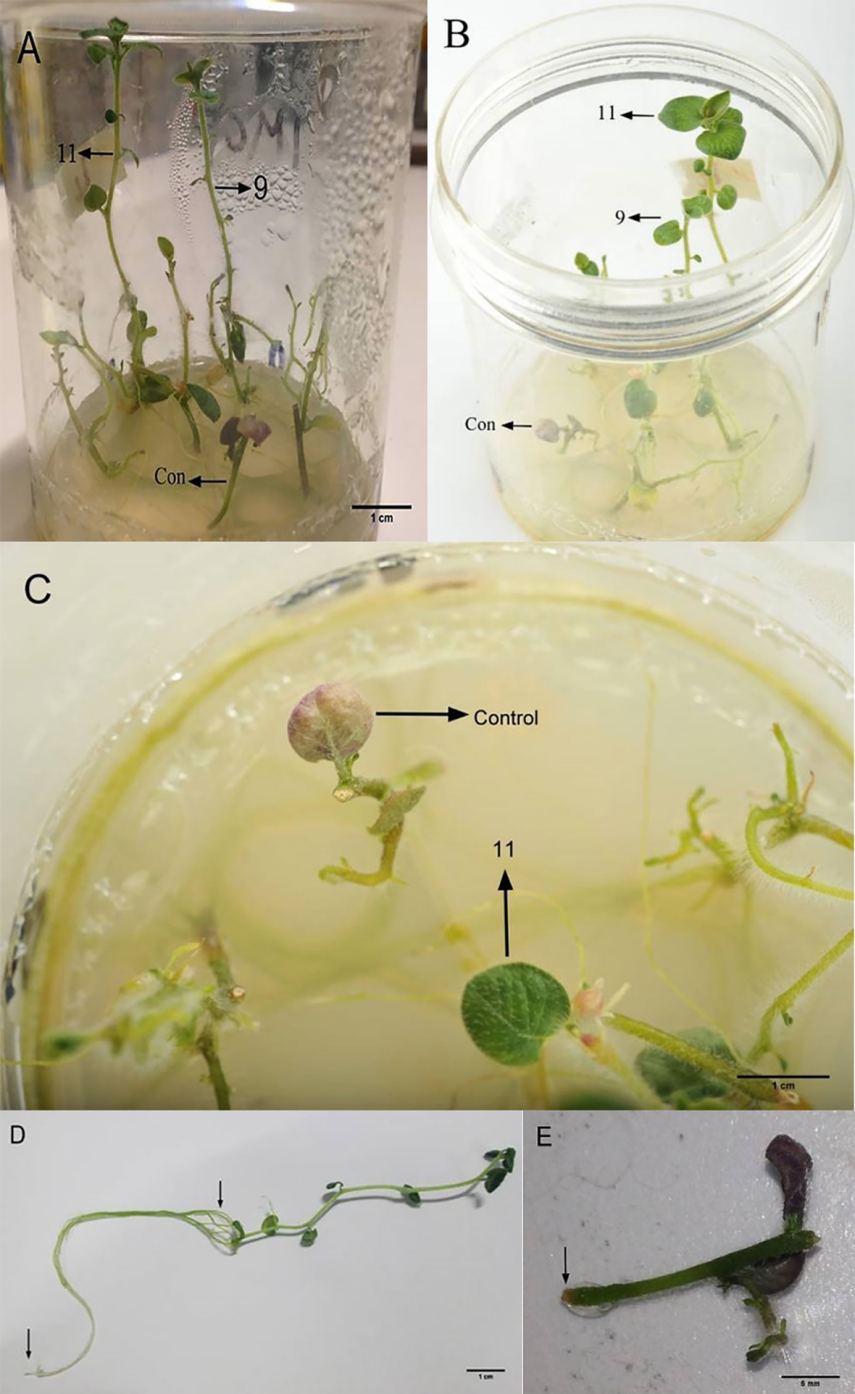
Growth of two *in vitro*-generated plant lines, namely L11 and L9, in the presence of 218 μM Cd compared to control. A&B: Comparison of shoot heights from 90^o^ and 45^o^ views, respectively; C: a healthy green leaf of a L11 plantlet and a necrotic leaf of a control shoot; D: a root of a L11 plantlet; E: lack of root formation in a control shoot segment.

**Fig 3:**
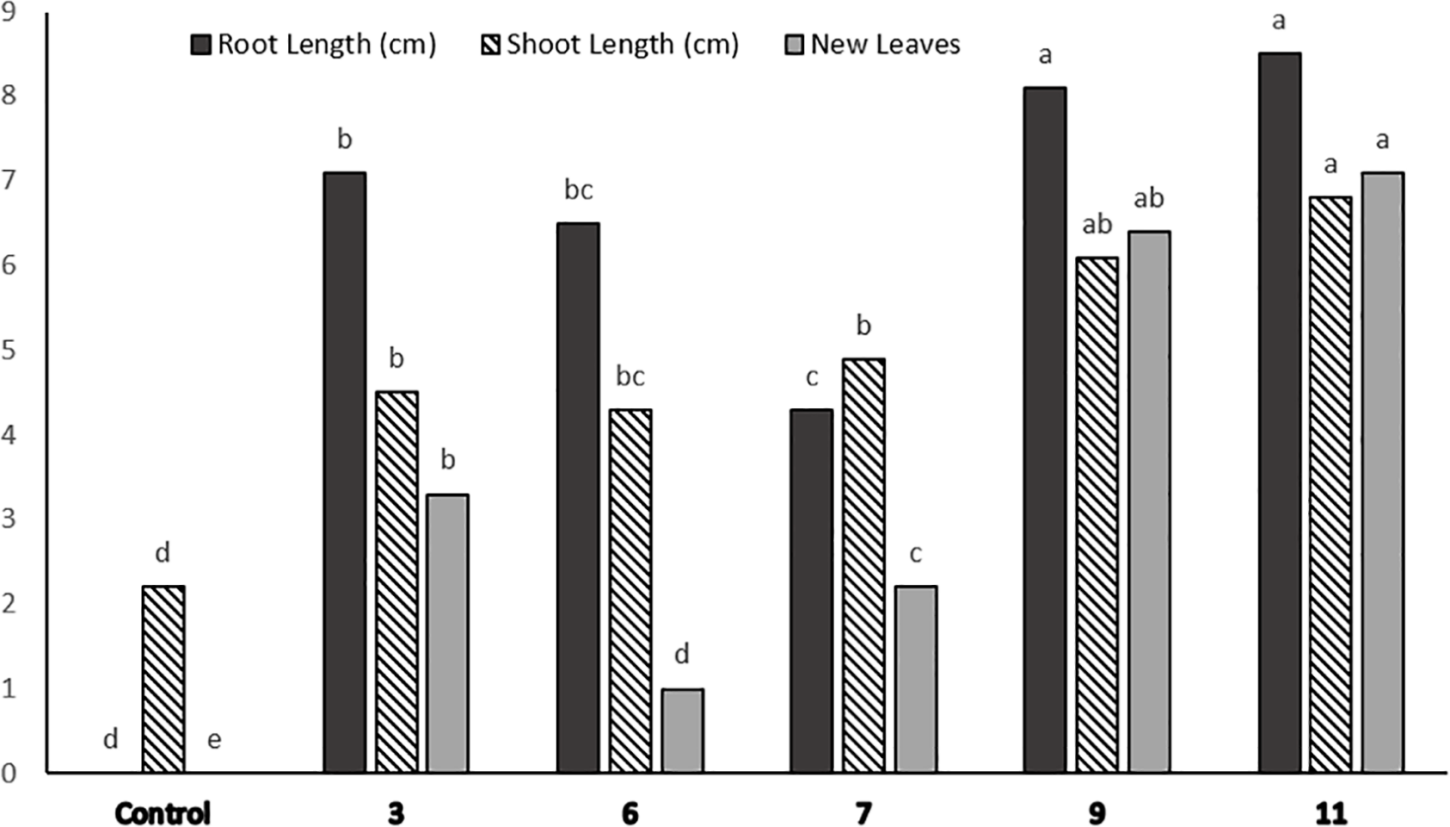
Differential growth responses (root length, shoot length and number of new leaves formed per plantlet) to 218 μM Cd of five Cd-selected potato lines selected from the first screening round after 20 days of culture. Means having the same letter are not significantly different by Duncan’s multiple range test (p<0.05).

To evaluate the five best performing lines further, they were subjected to the second round of screening under 218 μM Cd that was found to be lethal to control potato plantlets. The results of this screening round revealed that L11 and L9 not only survived this selection for 20 days, but interestingly also exhibited increased growth (Figs 2A, 2B and 3). The average root length of these two lines was more than 8 cm (Figs 2D and 3). In contrast, the control shoot cultures failed to form roots (Fig 2E). Likewise, shoot elongation was almost completely prevented in control shoot cultures, whereas the shoots of the plantlets in L11 and L9 were at least 4 cm long. Moreover, at least six new leaves were formed in the shoots of these two lines, while no new leaf was formed in the control shoot cultures (Fig 3).

**Fig 4:**
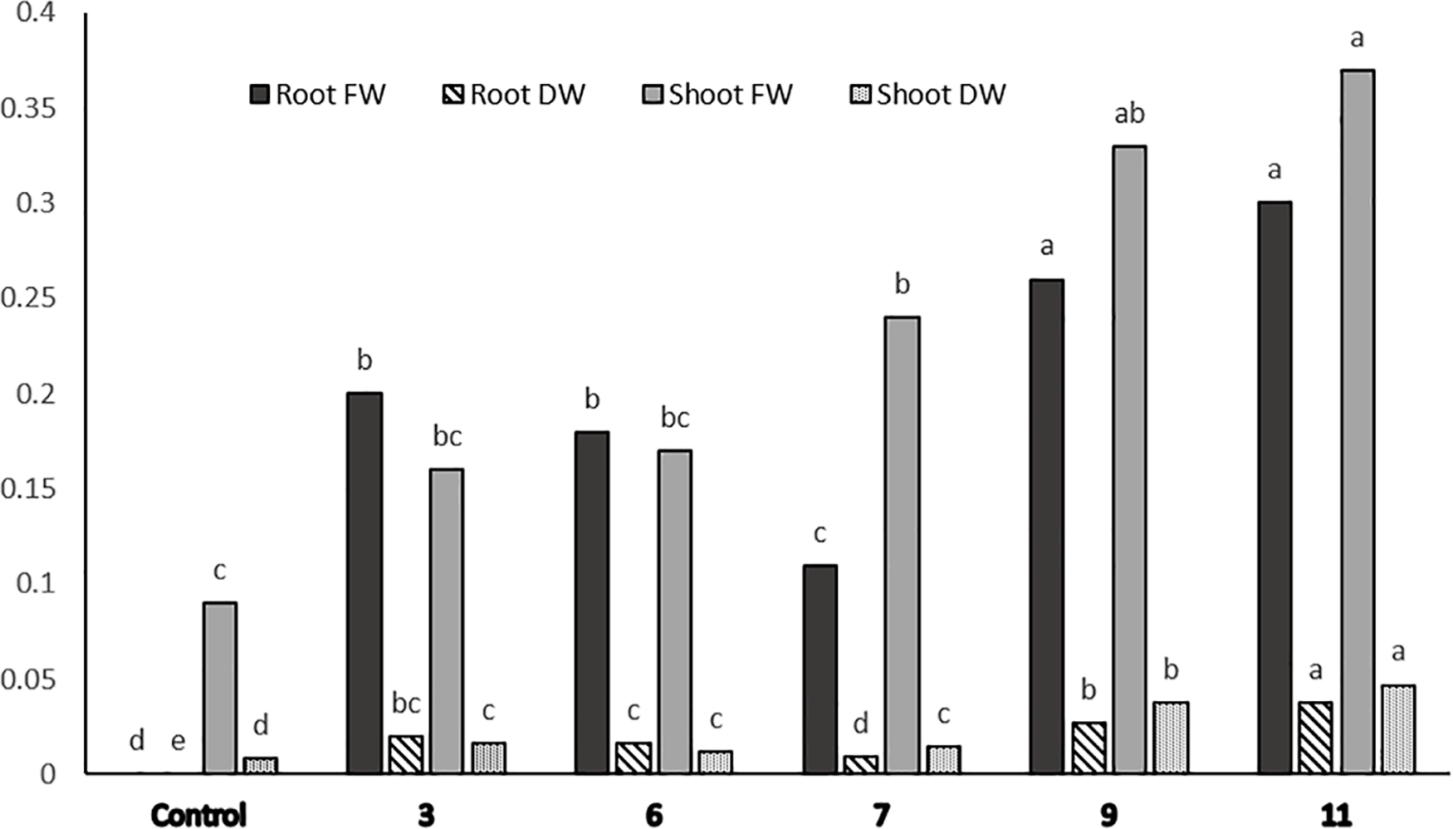
Differential responses to 218 μM Cd of 5 Cd-selected potato lines from the first screening round after 20 days of culture in relation to fresh and dry weights (FW and DW, respectively, in g per plantlet) of roots and shoots. Means having the same letter are not significantly different by Duncan’s multiple range test (p<0.05).

Apart from very few necrotic areas on the shoots, interestingly there was no major visible injury symptom on the L9 and L11 plantlets, whereas almost all the leaves in the control shoot cultures became completely necrotic in response to 218 μM Cd (Fig 2C and 2E). There was no difference in the fresh weights of the roots and shoots of these two lines but the dry root and shoot weights of L9 were 20 and 10% lower than those of L11, respectively (Fig 4).

### Biochemical analyses

**Fig 5:**
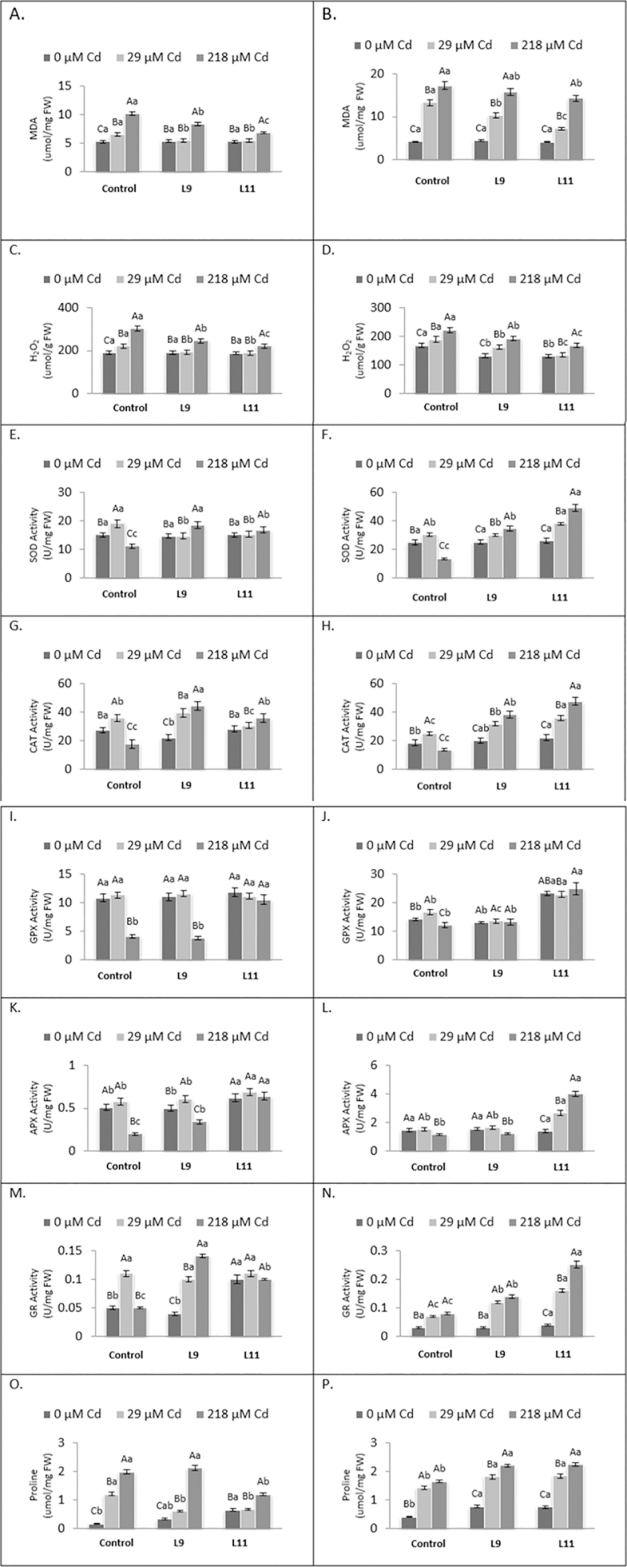
Levels of reactive oxygen species, antioxidative enzyme activities and proline in the leaves (left-hand panels) and roots (right-hand panels) of control, L9 and L11 plantlets exposed to 0, 29 and 218 μM Cd for 7 days. Upper and lower case letters are used for comparison among different treatments for the same line, and among different lines for the same treatment, respectively. Means with the same letter are not significantly different by Duncan’s multiple range test (p<0.05). The vertical bars show standard errors.

The MDA and H_2_O_2_ levels in the leaves and roots of control plantlets were increased with increasing levels of Cd in the medium (Fig 5A–5D). Those in the roots of L9 plantlets exhibited the same response. The MDA and H_2_O_2_ levels in the leaves of L9 and L11 plantlets were only increased in response to 218 μM Cd and there was no difference between the MDA and H_2_O_2_ levels in the plantlets grown in the medium with 0 or 29 μM Cd. The MDA level in the root of L11 plantlets was increased with increasing level of Cd in the medium but H_2_O_2_ level in the root of L11 was only increased in response to 218 μM Cd. The MDA and H_2_O_2_ levels in the leaves and roots of control were higher than those in L9 and L11 plantlets grown in media with 218 μM Cd.

The SOD and CAT activities in the leaves and roots of control plantlets were at the highest and lowest levels when grown in the presence of 29 and 218 μM Cd, respectively (Fig 5E–5H). In the roots of L9 and L11 plantlets, the SOD and CAT activities were increased with increasing levels of Cd in the medium but in the leaves they were only increased when the L9 and L11 plantlets were grown in medium with 218 μM Cd. Overall, the lowest SOD and CAT activities were found in the leaves and roots of control plantlets grown in medium with 218 μM Cd (Fig 5E–5H).

The GPX and APX activities were greatly reduced in the leaves of control and L9 plantlets grown in the medium with 218 μM Cd while there was no change in response to 29 μM Cd compared to 0 μM Cd (Fig 5I & 5K). These two enzyme activities in the leaves of L11 plantlets did not change when the plantlets were grown in increasing levels of Cd. The GPX and APX activities in the root of control, L9 and L11 plantlets generally varied slightly with increasing Cd levels in the medium except that there were bigger increases in APX activity in the root of L11 plantlets with increasing Cd levels in the medium (Fig 5J and 5L). The changes in GR activity in the leaves and roots of the control, L9 and L 11 plantlets were different (Fig 5M and 5N). Overall, in response to the leaves and roots of L11 plantlets had higher GPX, APX and GR activities than control plantlets.

In general, the levels of proline in the leaves and roots of control, L9 and L11 plantlets was increased with increasing levels of Cd in the medium except in the leaves of L11 plantlets where it only increased in response to 218 μM Cd (Fig 5O and 5P). The level of proline in the leaves of L11 plantlets was lower than that in control plantlets but it was higher than control in the root.

## Discussion

In the present study, 18 different *in vitro* selection treatments were designed taking into considerations of the following variables: early Cd exposure (started from callus induction stage) or during callus subculture and exposure duration (varied from 1 to 5 months). Plant regeneration from calli after lengthy stepwise *in vitro* selection could be difficult as there might be a loss of callus cell totipotency by DNA methylation or physical changes in chromosomes [27]. We were able to regenerate plants from all the 18 different *in vitro* selection treatments but the lowest plant regeneration frequency was found in the treatment 18 in which there was the highest number of callus subculture rounds (Table 1).

In the first round of post plant regeneration screening of the 18 different plant lines to evaluate Cd resistance compared to the control (plantlets used to start callus cultures for Cd selection), it was shown that Cd resistance exhibited at the callus level did not persist at the whole plant level in some plant lines. These lines were probably “escape” plants regenerated from the Cd-selected calli. High escape frequency is one of the criticisms on *in vitro* selection approach. For example, Vyver et al. [28] also reported a high escape rate of non-herbicide tolerant sugarcane plantlets selected under *in vitro* conditions. Both Cd-sensitive and Cd-resistant *Brassica juncea* plants were regenerated from Cd-selected calli [7]. Therefore, application of fast-screening method for eliminating escaped plants has been preferred to aiming for an escape-free selection system [29]. In the present study five out of the 18 putative somaclonal variant lines grew relatively better under Cd stress than control plantlets. Among these five lines, L11 and L9 were chosen for a closer examination due to their outstanding growth abilities under lethal Cd stress (218 μM Cd). In particular, the lack of any major visible injury on the root, shoot and leaf of L11 plantlets was very interesting. A lower biomass decrease in L11 compared to L9 in response to 218 μM Cd further differentiates between L11 and L9. It seems worthwhile to investigate in future studies the growth performance, tuber yield and Cd resistance of L11 plants under glasshouse and field conditions.

Measurement of lipid peroxidation and hydrogen peroxide production was useful in evaluating the extent of Cd-induced oxidative stress in the tissue culture-derived Cd-resistant potato lines (L9 and L11) compared to control plantlets. In response to Cd exposure, L11 showed the lowest level of Cd-induced oxidative stress compared to the control and L9. Activities of antioxidative enzymes in Cd-tolerant plants are often elevated in response to exposure to Cd [1]. Generally, antioxidative defence is one of the main strategies to alleviate the adverse impacts of oxidative stress in plants. Superoxide dismutase (SOD) is known as the first-line defender against reactive oxygen species (ROS) by scavenging superoxide radicals [30]. Catalase (CAT) and ascorbate peroxidase (APX) are the enzymes involved in removal of H_2_O_2_ produced by dismutation of superoxide radicals or non-enzymatic process in different cellular compartments. The balanced maintenance between ROS and antioxidative system is very critical for survival of plant cells under stress. Noticeably, the present analysis of L11 also confirmed the strong antioxidative defence system in the roots of L11 which was even able to function at the high Cd concentration (218 μM). The roots of L11 showed Cd dose-dependent increase in the activities of antioxidative enzymes, except GPX. This can be explained by the fact that both GPX and CAT enzymes independently catalyse the same reaction (reduction of hydrogen peroxide to water and O_2_) [31]. In contrast, activities of all the enzymes studied in control roots were inhibited. To some extents, the roots of L9 also exhibited a relatively stronger antioxidative defence system compared to control, but this was not as pronounced as the one seen in L11. Thus, the relative differential strengths of Cd resistance in L11 and L9 seem to be correlated with the antioxidative defence capabilities in coping with potential oxidative damages that could occur in plants under stress.

The leaves of the tissue culture-derived lines exhibited the same levels of most of the antioxidative enzymes studied here as those in the leaves of control when grown in the absence of Cd. L11 was exceptional in that their leaves showed higher APX and GR activities than control and this was correlated with higher Cd tolerance of L11 plantlets. Similar correlation was also found in a Cd-resistant cabbage cultivar [32] and transgenic potato plants overexpressing StDREB genes [33]. Activities of most of the antioxidative enzymes studied were inhibited in control leaves in response to 218 μM Cd, while they were less affected by this Cd level in the leaves of L9.

Increase in proline content has been known as one of the plant responses to heavy metal stress [34, 35]. Stress-resistant plant cultivars often have higher levels of free proline [36, 37]. Transgenic potato plants overexpressing StDREB (potato dehydration-responsive element binding protein gene) were also more resistant to Cd stress and exhibited a higher level of proline [33]. Unlike control leaves, both L9 and L11 were able to increase proline contents in their leaves in response to 218 μM Cd. The elevated accumulation of proline content in L9 and L11 compared to control plants could act as a molecular chaperon protecting protein integrity and enhancing the activities of different enzymes [34].

## Conclusion

The tissue culture-derived lines, particularly L11, seemed to have some mechanisms including the well-known enhanced participation of various antioxidative enzyme activities to mitigate the potential harm from stress-induced oxidative damaging effects better than control potato plantlets. It remains to be determined how well this novel potato line will perform under field conditions with varying levels of Cd contamination.
